# Computer-assisted analysis of pleural and subpleural lung ultrasound correlates with oxygenation in preterm infants

**DOI:** 10.1038/s41598-026-39333-6

**Published:** 2026-02-26

**Authors:** Selina K. X Zhang, Gillian W. C Foo, Sheryle R Rogerson, Niranjan Abraham, Penny Kee, Amir Zayegh, David G Tingay, Peter G Davis, Brett J Manley, Arun Sett

**Affiliations:** 1 Newborn Services, Joan Kirner Women’s and Children’s, Sunshine Hospital, Western Health, 176 Furlong Road, St Albans, Victoria, Melbourne, 3021 Australia; 2 Neonatal Research, Murdoch Children’s Research Institute, Victoria, Australia; 3 Newborn Research Centre, The Royal Women’s Hospital, Melbourne, Australia; 4 Department of Obstetrics, Gynaecology and Newborn Health, The University of Melbourne, Melbourne, Australia; 5 Department of Paediatrics, The University of Melbourne, Melbourne, Australia; 6 Department of Critical Care, The University of Melbourne, Melbourne, Australia; 7 Department of Paediatrics and Mercy Perinatal, The Mercy Hospital for Women, Victoria, Australia

**Keywords:** Preterm infants, Quantitative lung ultrasound, Grey-level co-occurrence matrix (GLCM), Textural analysis, Lung volume, Oxygenation, Diseases, Health care, Medical research, Physiology

## Abstract

Lung ultrasound (LUS) is increasingly used in neonatal intensive care, however visual scoring of LUS images remains subjective. Quantitative LUS (Q-LUS) is objective and whilst it detects small lung aeration changes in preterm animals, human data are limited. We hypothesised that Q-LUS would correlate strongly with oxygenation in very preterm infants born before 32 weeks’ gestation. We examined two quantitative image properties to assess whether Q-LUS correlates with oxygen status in very preterm infants: (1) mean-grey-value (MGV) and (2) second-order textural features (angular second moment, contrast, grey-level correlation (Q-LUS_correlation_), inverse difference moment, entropy). 560 LUS images were acquired from 70 very preterm infants using Venue 50 and Venue Go systems and analysed in Fiji ImageJ. Oxygenation was assessed using oxygen saturation index (OSI) and peripheral oxygen saturation to fraction of inspired oxygen (S/F) ratio. Spearman’s rank correlation coefficient (ρ) was used to determine correlations between Q-LUS measurements and infant oxygenation. We found that MGV correlated fairly with OSI (ρ=-0.46, [-0.68,-0.23], p<0.01) and S/F ratio (ρ=0.38, [95% CI: 0.16,0.61]; p<0.01), though results varied by ultrasound system (Venue 50 (OSI: ρ=-0.43, [-0.69,-0.18], p<0.01; S/F ratio: ρ=0.34, [0.06,0.63], p=0.02) and Venue Go (OSI: ρ=0.12, [-0.61,0.83], p=0.71; S/F ratio: ρ=-0.24, [-0.85,0.37], p=0.36). Among textural features, Q-LUS_correlation_ was moderately correlated with OSI (ρ=0.48, [0.25,0.71], p <0.01) and S/F ratio (ρ=-0.54, [-0.74,-0.34], p <0.01), and was consistent across systems. Overall, Q-LUS showed fair-to-moderate correlation with oxygenation in very preterm infants. Q-LUS_correlation_ was the only textural feature unaffected by machine type, supporting its potential as a machine-independent, indirect measure of oxygenation.

**Supplementary Information:**

The online version contains supplementary material available at 10.1038/s41598-026-39333-6.

## Introduction

Effective lung-protective ventilation strategies are essential in mitigating ventilator-induced lung injury (VILI)^1^. VILI is a key contributor to bronchopulmonary dysplasia, a chronic disease that affects preterm infants and shows minimal improvement in respiratory outcomes over time^2,3^. In conditions associated with atelectasis, lung-protective ventilating strategies aim to deliver effective gas exchange for preterm infants whilst reducing lung injury by minimising delivered tidal volumes while recruiting and stabilising atelectatic lung units through an open lung ventilation approach^1^. This requires tools which provide reliable, real-time estimation of lung aeration at the bedside. Currently, the only method that is routinely used to assess lung aeration in neonates at the bedside is chest X-ray (CXR). CXR, however, exposes neonates to ionising radiation and provides inaccurate lung aeration measurements, particularly in preterm infants^4,5^. In order to deliver lung-protective ventilation, techniques that provide accurate, real time, radiation-free assessments of lung aeration are required.

A suitable alternative may be analysis of pleural and subpleural regions of lung ultrasound (LUS) images. LUS is a point of care, radiation-free modality that can accurately diagnose common neonatal respiratory disorders such as meconium aspiration syndrome, respiratory distress syndrome, transient tachypnoea of the newborn and pneumothorax^6,7^. LUS also has functional applications; Brat et al. demonstrated a moderate correlation between LUS aeration score and multiple oxygenation indices (transcutaneous partial pressure of oxygen (Ptco_2_) to fraction of inspired oxygen (Fio_2_) ratio, alveolar-arterial gradient, oxygenation index, and arterial to alveolar ratio) in preterm infants^8^.Similarly, we demonstrated that LUS aeration score was able to detect large changes in total and regional lung aeration in real-time and correctly identified opening and closing pressures in preterm lambs^9^. In the present study, we specifically focused on the pleural and immediately subpleural image regions, rather than the entire lung field. This decision was supported by prior work from Raimondi et al. who demonstrated that ultrasound-derived features originating at the pleural–lung interface are sensitive to pressure- and volume-mediated changes in lung aeration, even when analysis is restricted to subpleural regions^10^. Accordingly, we tailored our region of interest to this anatomically relevant zone. Importantly, our approach is not intended to automatically detect all lung ultrasound artefacts, but rather to quantify aeration-related changes within this defined pleural/subpleural ROI. These prior studies used scoring systems that are assigned visually by the operator and are based on categorical ultrasound artefact patterns^8,9^. However, visual scoring lacks the precision required to detect small changes in lung aeration, likely due to the limited ability of categorical artifact based systems to discern subtle changes that occur at lower volume states, and may be subjective as it is based on user-interpretation^11^.

A potential solution is computer-assisted analysis of the pleural and subpleural region of LUS images through the calculation of a mean greyscale value (MGV) and second-order textural features^12,13^. MGV reflects the average pixel intensity within a defined region of interest, thereby capturing overall image brightness. By capturing the full greyscale spectrum within the pleural and immediately subpleural region, MGV provides a simple yet sensitive measure of aeration, making it well suited to detect small, real-time changes in lung aeration on LUS. In a study involving 40 preterm lambs, we previously demonstrated that MGV could detect changes in total and regional lung aeration and provided an objective quantification of LUS images^12,14^.

Image analysis can also quantify textural features, an intrinsic property of virtually all surfaces and plays a key role in identifying objects or regions of interest within an image^13^. Texture, traditionally described qualitatively as the “smoothness” or “roughness” of a surface, can instead be expressed through discrete numerical measures derived from second-order statistical calculations. These textural features include angular second moment, contrast, correlation (Q-LUScorrelation), inverse difference moment, and entropy. This is done by formulating a grey-level co-occurrence matrix (GLCM) that quantifies how frequently different intensity levels (grey levels) occur next to each other in an image^13^. Textural analysis is increasingly applied in cancer radiomics, computed tomography and magnetic resonance imaging to differentiate between tissue types and pathological states, offering structural information that may not be discernible through visual inspection alone^15,16^.

We hypothesised that computer-assisted image analysis of the pleural and immediate subpleural region of LUS images would strongly correlate with oxygenation in very preterm infants born before 32 weeks’ gestation. To address this hypothesis, we analysed LUS images from 70 very preterm infants that had been evaluated using visual inspection alone in a previously published study^5^.

### Aims

- Primary aim: to assess the correlation between MGV and infant oxygenation indices, specifically the oxygen saturation index (OSI) and S/F ratio, in very preterm infants.
- Secondary aim: to evaluate whether individual GLCM features - including angular second moment, contrast, correlation (Q-LUS_correlation_), inverse difference moment, and entropy, correlate with oxygenation in very preterm infants.

## Methods

This is a sub-study of a recently published report exploring the relationship between CXR and LUS images of the preterm infant lung^5^. The original study was performed at The Royal Women’s Hospital and Joan Kirner Women’s and Children’s, Sunshine.

Hospital, Melbourne, Australia between 2020 and 2022. Informed prospective parental consent was obtained for all infants and the study was approved by The Royal Children’s Hospital Human Research Ethics Committee, Melbourne, Australia (Approval Number: HREC/73509/RCHM-2021). All methods were performed in accordance with the relevant guidelines and regulations. Ethics approval was obtained for this sub-study prior to completion of the original study.

In this sub-study, de-identified LUS images from the original dataset were used for computer-assisted image analysis to measure the (1) MGV and (2) textural features of the image. These quantitative image properties were then correlated with the following oxygenation indices (oxygen saturation index (OSI) and SpO_2_ to FiO_2_ ratio (S/F ratio) to determine their association with very preterm infant oxygenation status.

### Subjects

The study sample consisted of 70 very preterm infants who were >24 hours of age and were undergoing a clinically indicated CXR^5^. Clinical demographics were as reported in the original study (Supplementary Table 1). The study population included some infants on no respiratory support and also some receiving nasal high-flow, nasal continuous positive airway pressure (CPAP), conventional mechanical ventilation and high-frequency oscillatory ventilation. Infants were also only eligible if LUS could be performed <1 hour before the planned CXR.

Exclusion criteria included infants with known congenital lung malformations, congenital diaphragmatic hernia, major congenital heart defects, pleural effusion, pneumothorax or who were soon to receive exogenous surfactant.

### LUS image acquisition

LUS images were acquired from the original dataset. These images were obtained using Venue Go and Venue 50 ultrasound systems (GE Healthcare) with a high-frequency hockey-stick linear transducer. Depth was set to 3 cm, and the focal zone was positioned at the pleural line. Ultrasound setting and technique were as per the original study^5^. Eight sections were imaged: left anterior lower, left anterior upper, left lateral lower, left lateral upper, right anterior lower, right anterior upper, right lateral lower, right lateral upper. For each infant, quantitative measurements derived from the eight lung ultrasound images were then averaged to generate a single representative Q-LUS value, which was then compared with oxygenation indices measured at the time of imaging.

### Quantitative image analysis

**Fig. 1:**
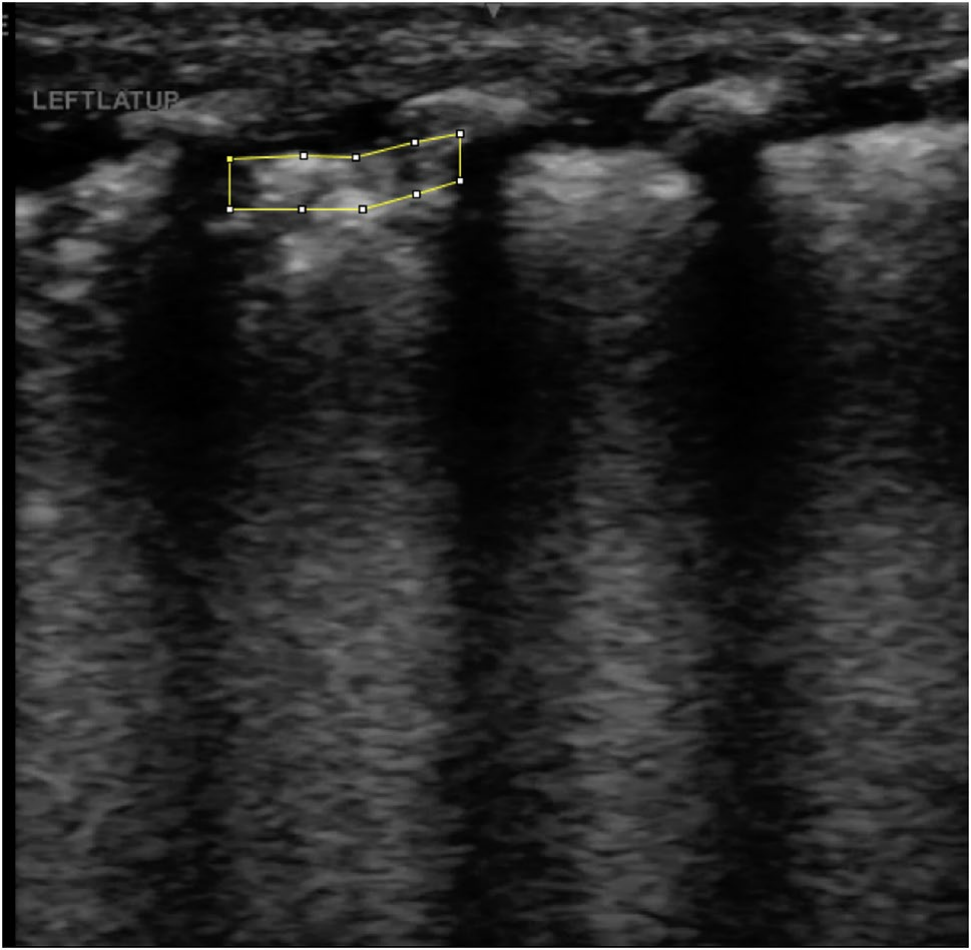
Manual selection of pleural region of interest on lung ultrasound.

LUS images were then de-identified and exported in uncompressed DICOM format from both ultrasound systems and converted to 8-bit format in Fiji (Image J) for quantitative image analysis. The 8-bit conversion enabled quantisation of pixel intensities across a standardised 256-level greyscale range (0–255), which was necessary for GLCM computation. No additional compression or preprocessing was applied. The pleural and immediate subpleural region of interest (ROI) was manually delineated by one investigator who was unaware of the clinical status (SZ) (Fig. 1). The pleural ROI was manually delineated on de-identified LUS images using Fiji (ImageJ). The superior boundary was defined by the pleural surface, the lateral boundaries by adjacent rib shadows, and the inferior boundary by a fixed depth of 50 pixels (approximately 1 mm). This ROI was then used for subsequent greyscale and texture analyses to calculate the MGV and grey-level textural features, which are summarised and defined in Table 1. Further methodological details are provided in the Supplementary Information.

**Table 1:** Overview of quantitative lung ultrasound (LUS) parameters analysed in this study.

**Parameter**	**Type**	**Range**	**Computation**	**Interpretation**
Mean grey value (MGV)	Absolute	0–255 (8-bit)	Average pixel intensity within ROI	Higher values indicate brighter image
Angular second moment (ASM)	Absolute	0–1	Sum of squared GLCM probabilities	Higher values indicate greater image homogeneity
Contrast	Absolute	≥0	Weighted sum of squared intensity differences between neighbouring pixels	Higher values indicate greater local intensity variation
Correlation (Q-LUS correlation)	Relative	−1 to 1	Linear dependency of grey levels on neighbouring pixels	Higher values indicate structured, predictable patterns
Inverse difference moment (IDM)	Absolute	0–1	Sum of GLCM values weighted by inverse of intensity difference	Higher values indicate smoother texture
Entropy	Absolute	≥0	Negative sum of GLCM probabilities multiplied by their logarithm	Higher values indicate greater texture randomness

The table summarises each metric, indicates whether values are absolute or relative, briefly describes how each parameter is computed, and outlines its intended interpretation based on pleural and subpleural image analysis.

### Indices of oxygenation

Indices of oxygenation used in our study were the (1) oxygen saturation index (OSI) and (2) SpO_2_ to FiO_2_ ratio (S/F ratio). These indices were extracted from the original study and were calculated from the peripheral oxygen saturation (SpO_2_), measured by pulse oximetry, FiO_2,_ and respiratory support parameters recorded at the time of lung ultrasound imaging^5^.

1. OSI

OSI was determined by the following equation:

$$\left( {OSI = \;mean\;airway\,pressure\;\left( {MAP} \right)*\frac{{FiO_{2} }}{{SpO_{2} }}} \right)$$

MAP is the mean airway pressure (in centimetres of water, cmH_2_O) measured at the airway opening^17^. The OSI was calculated as the product of MAP and FiO₂, divided by SpO₂. The OSI was utilised because routine arterial blood gas analysis was not performed. In infants receiving CPAP, the set continuous positive airway pressure was chosen to be the MAP^17^.

(2) SpO_2_ to FiO_2_ ratio (S/F ratio)

In infants who were not receiving respiratory support or were receiving nasal high flow, in whom MAP was not measured or available, S/F ratio was used to quantify oxygenation^18^.

## Statistical analysis

The sample size was restricted to that recruited to the original study.(9) Histograms were constructed for OSI and S/F ratio to determine distribution of data. Spearman’s rank correlation coefficient was used to assess the relationship between OSI and S/F ratio, with MGV, ASM, contrast, Q-LUS_correlation_, IDM and entropy. Strength of correlation was defined according to the criteria outlined in the original study as follows: weak, Spearman’s rho (ρ)< 0.30; fair, ρ=0.30-0.49; moderate, ρ=0.50-0.69; and strong, ρ>0.70 with 95% confidence intervals (CI)^5^. Statistical significance was defined as p<0.05. Scatter plots were used to visualise the associations between oxygenation indices, MGV, and other textural features. Analysis was performed using R (R: A Language and Environment for Statistical Computing)^19^.

## Results

**Fig. 2:**
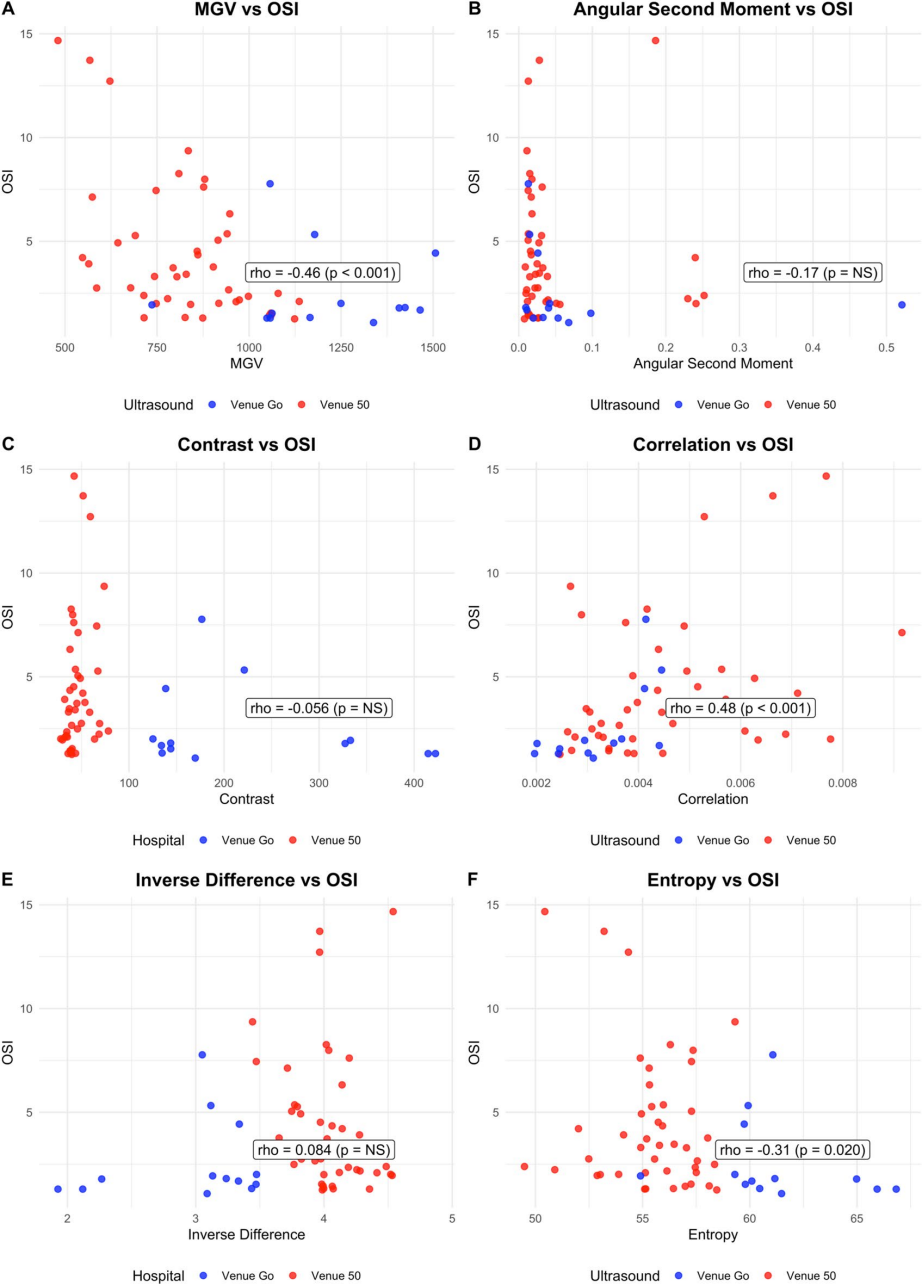
Correlation of MGV and textural features with OSI. Individual dots represent individual image analysis and OSI pairs. Images acquired from a Venue Go (blue) and Venue 50 (red) ultrasound system. Higher OSI values reflect worsening oxygenation.

**Fig. 3:**
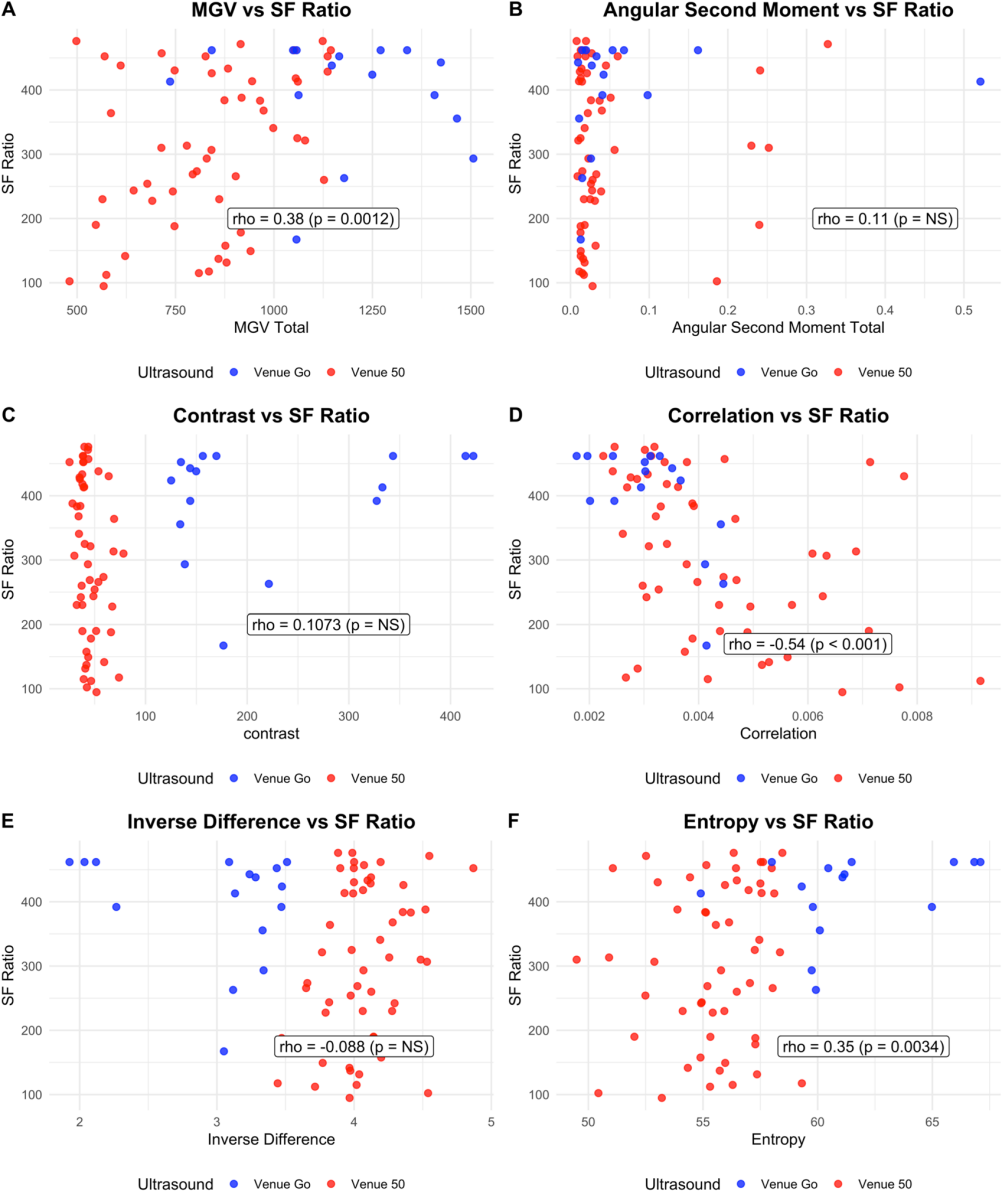
Correlation of MGV and textural features with S/F ratio. Individual dots represent individual image analysis and S/F ratio pairs. Images acquired from a Venue Go (blue) and Venue 50 (red) ultrasound system. Higher S/F ratio values reflect improving oxygenation.

Patient characteristics are described in the original study (Supplementary Table 1)^5^. A total of 560 LUS images were analysed from 70 infants. Correlation of MGV and GLCM measurements with OSI and S/F ratio across ultrasound-machine subgroups are summarised in Table 2**.** A comprehensive table reporting all correlations, including non-significant results, is provided in the Supplementary Material (Supplementary Table 2). Scatter plots of this relationship are shown in Figures 2 and 3**.**

**Table 2:** Spearman’s correlation and 95% confidence intervals (CI) for grey level co-occurrence matrix (GLCM) features with oxygen saturation index (OSI) and saturation to fraction of inspired oxygen (S/F) ratio.

Oxygenation	Overall		Venue 50		Venue Go	
	OSI (95% CI)	S/F Ratio (95% CI)	OSI (95% CI)	S/F Ratio (95% CI)	OSI (95% CI)	S/F Ratio (95% CI)
MGV	-0.46 (-0.68,-0.23)	0.38 (0.16,0.61)	-0.43 (-0.69,-0.18)	0.34 (0.06,0.63)	NS	NS
ASM	NS	NS	NS	NS	NS	NS
Contrast	NS	NS	0.38 (0.13,0.62)	-0.31(-0.52,-0.10)	NS	NS
Correlation	0.48 (0.25,0.71)	-0.54 (-0.74,-0.34)	0.33 (0.01,0.64)	0.45 (-0.72,-0.18)	0.65 (0.14,0.94)	-0.62 (-1.05,-0.25)
IDM	NS	NS	-0.33 (-0.62,-0.03)	NS	NS	NS
Entropy	-0.31(-0.58,-0.03)	0.35 (0.12,0.57)	NS	NS	-0.58 (-1.0,-0.13)	NS

### MGV and oxygenation

MGV was fairly correlated with both OSI (ρ=-0.46 [-0.68,-0.23], p<0.01) and S/F ratio (ρ=0.38 [95% CI 0.16,0.61]; p<0.01). There were ultrasound-specific differences in results. Images from the Venue 50 ultrasound machine showed moderate correlation between MGV and oxygenation (OSI: ρ=-0.43, [-0.69,-0.18], p<0.01; S/F ratio: ρ=0.34, [0.06,0.63], p=0.02). Correlation for images acquired from the Venue Go ultrasound machine was not significant (OSI: ρ=0.12, [-0.61,0.83, p=0.71; S/F ratio: ρ=-0.24, [-0.85,0.37], p=0.36).

### Grey-level textural features and oxygenation

Among textural features, Q-LUS_correlation_ and entropy demonstrated statistically significant relationships with oxygenation. Q-LUS_correlation_ showed a fair positive relationship with OSI (ρ=0.48 [0.25,0.71], p-value <0.01) and a moderate negative association with S/F ratio (ρ=-0.54 [-0.74,-0.34], p-value <0.01). Entropy was fairly correlated with both indices (OSI: ρ=–0.31 [-0.58,-0.034], p=0.02; S/F ratio: ρ=0.35, [0.12,0.57], p<0.01). ASM, contrast, and IDM were not significantly associated with oxygenation (*p*>0.05).

Images obtained from the Venue 50 ultrasound machine demonstrated that contrast, Q-LUS_correlation_ and IDM were fairly associated with OSI (contrast: ρ=0.38, [0.13,0.62], p=0.01; Q-LUS_correlation_: ρ=0.33, [0.01,0.64], p=0.03; IDM: p=-0.33, [-0.62,-0.03], p=0.03). Contrast and Q-LUS_correlation_ were fairly associated with S/F ratio (contrast: ρ=–0.31, [-0.52,-0.10], p=0.02; Q-LUS_correlation_: ρ=0.45, [-0.72,-0.18], p< 0.01).

Images from the Venue Go ultrasound machine showed that Q-LUS_correlation_ was strongly associated with OSI and S/F ratio (OSI: p=0.65, [0.14,0.94], p=0.02; (S/F ratio: p=-0.62; [-1.05,-0.25], p=0.01). Entropy was moderately correlated with OSI (p=-0.58, [-1.0,-0.13], p=0.03).

Correlations are highlighted as fair (orange) or moderate (green). All results statistically significant (p<0.01) unless otherwise stated. ASM = angular second moment; IDM = inverse difference moment; MGV = mean gray value; NS = not significant.

## Discussion

There are three key findings of the present research. Firstly, Q-LUS_MGV_ was fairly correlated with oxygenation, where higher MGV values correspond to better oxygenation (lower OSI; higher S/F ratio). Although this is consistent with previous findings that LUS aeration scores correlates with oxygenation^5^, contrary to our hypothesis, Q-LUS_MGV_ did not demonstrate stronger correlation than visual scoring. In addition, quantitative analysis was restricted to a single, predefined subpleural region of interest within one lung ultrasound window; therefore, Q-LUS reflects regional subpleural echogenicity and may not capture the full spatial heterogeneity of lung ultrasound artefacts or represent global lung aeration. A possible explanation is that Q-LUS_MGV_ was derived from an ROI limited to the pleural and immediate subpleural zone. As a result, key sonographic artefacts that indicate different lung aeration states, such as A-lines, B-lines, and subpleural consolidations were not fully captured. In contrast, semi-quantitative visual scoring incorporates a wider range of features across the lung field, including these artefacts, which may provide a more complete assessment of lung aeration and its correlation with oxygenation^8,20^. This is supported by Raimondi et al. who found that in a neonatal cohort, visual LUS aeration scoring outperformed computer-assisted greyscale analysis in evaluating respiratory status, despite both methods demonstrating correlation with clinical parameters^10^. These findings reinforce the value of artefact interpretation in visual LUS analysis.

This finding, however, should be distinguished from conditions in which pleural line appearance is directly altered by disease. For example, Brusasco et al. successfully differentiated acute respiratory distress syndrome from cardiogenic pulmonary oedema using greyscale analysis of the pleural and subpleural space^21^, the same region of interest used in the present study. In their study, significant findings were likely driven by distinct morphological changes at the pleural surface, whereas differences in oxygenation alone may not produce similarly visible structural alterations. Future studies analysing a larger ROI extending beyond the pleural line may help improve the correlation between Q-LUS_MGV_ and oxygenation indices. A key challenge in this approach will be differentiating between hyperechoic intensity values arising from A-lines and B-lines, as both appear bright on greyscale imaging but represent different states of lung aeration. Moreover, while a well-defined and bright pleural line is often associated with adequate lung aeration, increased brightness alone does not always indicate complete aeration. In fact, a thickened and more hyperintense pleural line can also be a sign of pathology**,** such as in respiratory distress syndrome^22^. Therefore, future advances in Q-LUS must account not only for the depth and extent of the ROI but also for the nuanced interpretation of echogenic patterns, to more accurately reflect underlying lung pathology.

Our second finding was that there were ultrasound-machine specific differences in results despite standardising image acquisition protocols in the original study. This may be attributed to inherent variability in system-specific image processing. Quantitative greyscale values are highly dependent on the ultrasound system, probe, and imaging settings, underscoring the need for standardisation in LUS image acquisition^20^. Likewise, previous studies have emphasised the role of ultrasound settings in LUS image acquisition, showing that different machines can produce varying findings^23^. For instance, different ultrasound settings may variably depict features such as shred signs, pleural effusions, or consolidations, which can lead to over or underestimation of disease severity. These artefactual differences may inadvertently influence clinical interpretation, particularly in conditions like respiratory distress syndrome or pulmonary haemorrhage, where imaging features often overlap.

Ultrasound images may also vary significantly between operators. Operators modify parameters such as gain, depth, focus, or dynamic range based on their training, clinical judgement or institutional protocols. These adjustments can significantly alter the visual appearance of LUS images, particularly in terms of brightness and contrast, which in turn, influences the quantification of textural features. While the Q-LUS_correlation_ feature remained relatively robust to such variations due to its reliance on relative pixel intensity relationships rather than absolute values, other metrics may be more sensitive to operator-dependent factors. Thus, these sources of variability highlight the critical need for standardising both ultrasound machine settings and operator techniques in multi-centre studies to ensure consistent, reproducible, and clinically meaningful LUS-based assessments. Without rigorous standardisation of both machine protocols and operator techniques, observed differences may reflect artefactual variability rather than true physiological changes.

Finally, among the textural features analysed, Q-LUS_correlation_ emerged as the most reliable predictor of oxygenation across different ultrasound machines. It showed a fair correlation with oxygenation in images acquired from the Venue 50 ultrasound machine and a moderate correlation in images acquired from the Venue Go machine. Importantly, this indicates that Q-LUScorrelation reflects physiologically meaningful differences in lung aeration rather than simply differences in ventilator settings, because in spontaneously breathing preterm infants receiving non-invasive respiratory support, ventilator-set airway pressure does not reliably reflect either global or regional lung volume.

Furthermore, there was no clustering of Q-LUS_correlation_ measurements based on ultrasound system, suggesting independence from machine-specific image processing characteristics. A likely explanation for this is that unlike other textural features that measure absolute pixel intensity, Q-LUS_correlation_ is a *relative* measure of texture patterns. This likely overcomes the inherent differences between ultrasound systems that occur despite standardising user settings, image acquisition parameters and consequential changes in brightness and image characteristics - factors that are known limitations of LUS aeration scoring^23^. This finding is important as it highlights the potential of Q-LUS_correlation_ to serve as a consistent and machine-independent method to objectively analyse LUS images, suitable for use across different clinical settings and ultrasound platforms. Together, these findings indicate that MGV and other absolute textural features may have limited utility in multicentre studies without machine-specific calibration, whereas Q-LUScorrelation offers greater promise for cross-platform clinical application.

Our study has limitations. Firstly, GLCM analysis was only performed in one direction (0 degrees) rather than all four axes. This meant that only left-to-right pixel intensities were analysed, which is only a partial representation of the true textural features in the image, potentially missing important vertical or diagonal features. Since LUS artifacts such as B-lines are oriented perpendicular to the pleural line, limiting analysis to the horizontal axis may underrepresent critical aspects of image texture relevant to lung aeration and pathology. However, Q-LUS_correlation_ still demonstrated consistent associations with oxygenation across two independent hospital cohorts and different ultrasound machines**.** Future research should focus on analysing Q-LUS_correlation_ using multi-directional GLCM analysis to determine whether it provides more robust insights into the relationship between image texture and oxygenation.

Delineation of the subpleural ROI was performed retrospectively rather than in real time, limiting immediate bedside translation and introducing a degree of operator dependence. In addition, ROI selection was undertaken by a single investigator, raising the possibility of selection bias, particularly when identifying regions intended to represent the worst aeration state while excluding rib artefacts. These factors have implications for reproducibility, scalability, and future clinical implementation. However, selection of an appropriate pleural ROI requires a level of expertise that is achievable within a short learning curve for lung ultrasound^24^, and excellent intra-observer agreement for manual subpleural ROI delineation has been demonstrated in controlled experimental settings using the same analysis pipeline (intra-class correlation coefficient = 0.87–0.93). Nonetheless, reliance on offline, expert-driven ROI selection may limit reproducibility and scalability in routine clinical practice.

Although lung aeration is known to be regionally heterogeneous, we did not perform region-specific analyses or evaluate multiple ROIs per image in the present study, as our primary aim was to establish feasibility and physiological relevance in a proof-of-concept validation rather than to characterise regional aeration patterns. Future studies incorporating multiple ROIs, especially in automated analysis pipelines, will be important to evaluate the impact of ROI selection and regional heterogeneity. This is particularly so given that prior evidence has shown that lung ultrasound can detect regional aeration inhomogeneity in ventilated preterm lungs^14^.

While automated segmentation may improve scalability, such approaches may also be more susceptible to artefacts and image noise and will therefore require careful validation^25^.

Moreover, only approximations of lung aeration (OSI and S/F ratio) were used as markers of oxygenation. Oxygenation can be influenced by non-respiratory factors such as haemoglobin status and cardiovascular function^26,27^, making these indices imperfect comparators. This limitation may partly explain the weaker correlations observed between Q-LUS features and oxygenation, and suggests that LUS could in fact be more reliable than OSI or S/F ratio for assessing oxygenation. Future studies should aim to validate Q-LUS against gold-standard measures of lung aeration, However, gold-standard measures such as computed tomography and whole-body plethysmography are not routinely used in clinical practice because of radiation exposure or limited feasibility in infants.

Finally, this study was a substudy, which limited the design flexibility and statistical power to detect smaller but clinically meaningful effects. These factors should be considered when interpreting the findings and highlight the need for confirmation in larger, prospectively designed studies.

## Conclusion

Computer-assisted analysis of LUS images is fairly-to-moderately correlated with oxygenation in very preterm infants, but not more so than visual assessment of LUS images using semiquantitative scoring. Among the greyscale textural features evaluated, only Q-LUS_correlation_ was consistently reliable across different ultrasound machines and hospital settings. This finding suggests its potential utility as an objective and machine-independent metric for assessing oxygenation status in very preterm infants.

## Supplementary Information

Supplementary Information.

## Data Availability

The datasets analysed during this study are not publicly available due to restrictions under the approved ethics protocol. Although all images were de-identified, ethics approval and parental consent limit data use to the approved study. De-identified data may be accessed from the corresponding author on reasonable request and with appropriate ethics approval.

## Abbreviations

ASM: Angular second moment
BPD: Bronchopulmonary dysplasia
cm H_2_O: Centimetres of water
CPAP: Continuous positive airway pressure
CXR: Chest X-ray
CI: Confidence interval
FiO_2_: Fraction of inspired oxygen
GLCM: Grey-level co-occurrence matrix
NHF: Nasal high-flow
IDM: Inverse difference moment
LUS: Lung ultrasound
MAP: Mean airway pressure (cmH₂O)
MGV: Mean grey value
OSI: Oxygen saturation index
ρ: Spearman’s rho
ROI: Region of interest
S/F ratio: Saturation to fraction of inspired oxygen ratio
SpO_2_: Peripheral oxygen saturations (%)
Q-LUS: Quantitative lung ultrasound
VILI: Ventilator induced lung injury
