# Supplementary material for: Computer-assisted analysis of pleural and subpleural lung ultrasound correlates with oxygenation in preterm infants
Source: Sci Rep. 2026 Feb 26;16:11103. doi: 10.1038/s41598-026-39333-6 (PMC13043698; doi:10.1038/s41598-026-39333-6)
Supplement: Supplementary file 1 — Supplementary Information. [file 41598_2026_39333_MOESM1_ESM.docx]

**Supplementary Information**

**Supplementary methods:**

Textural features were calculated using Fiji (Image J) using second-order statistical analysis of texture. This process examines the spatial relationships between grey-level intensities of pixels within an image. More specifically, it finds the probability of finding a pair of pixels with specific intensity values in a given spatial relationship. This is done by formulating a grey-level co-occurrence matrix (GLCM) that quantifies how frequently different intensity levels (grey levels) occur next to each other in an image[12].

For example, consider an image composed of four shades ranging from 0 to 3 in intensity, with the value 0 representing black, the value 3 representing white, and the values 1 and 2 as shades of grey (**Supplementary Figure 1(a)**). The GLCM examines pixel relationships in four standard directions**: 0° (left to right), 45° (top left to bottom right), 90° (top to bottom), and 135° (top right to bottom left) (Supplementary Figure 1(b)). Mathematically, this relationship can be described as C(i, j), where ‘i’ is the row and ‘j’ is the column within the correlation matrix. The function C(i, j) would then represent how many times a pixel of intensity ‘j’ occurs in a particular direction of intensity ‘i’.**

**
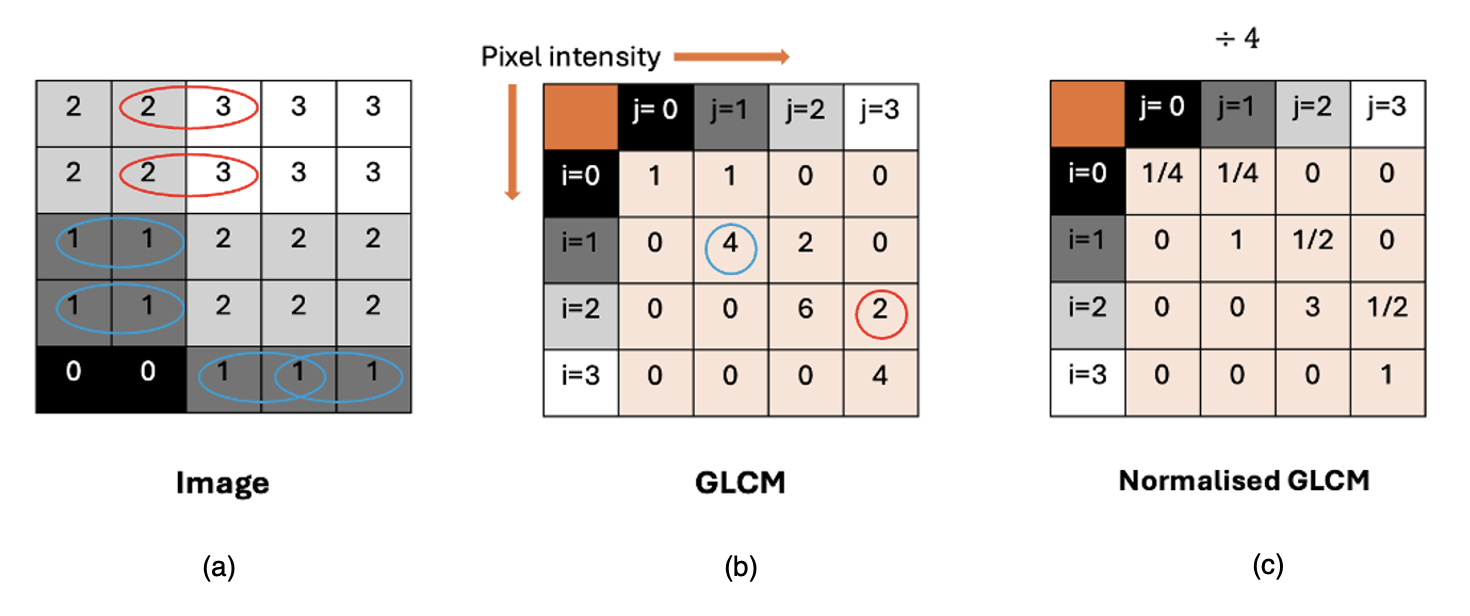
**

**Supplementary Figure 1.** Example of GLCM generation and normalisation from a greyscale image. ***Fiji (Image J) software analyses pixel relationships in directions of 0°, 90°, 180° and 270°.* (a)** Example of an image with 4 shades ranging from black (0) to white (3). Co-occurrence pairs of C(2,3) and C(1,1) are highlighted in red and blue respectively. These are then represented in a co-occurrence matrix **(b)** and normalised based on the total number of pixels **(c)**. Statistical operations are performed based on the direction of pixel relationships in four standard directions.

To simplify this example, assume a GLCM calculation considered pixel relationships at ***0°* only. This would compute a matrix that counts how many times a pixel with intensity ‘i’ is next to a pixel with intensity ‘j’ in the *left -to-right* direction. For example, if we wanted to compute C(2,3), this would be the sum of how many times a pixel of intensity 3 occurs to the right of a pixel with intensity of 2. From Supplementary Figure 1(a), we can determine this would be twice (C(2,3) = 2). Similarly, for a pixel with intensity 1, another pixel of intensity 1 (C(1,1) appears to the right of it four times in total.**

These directional matrices are then averaged to produce a single, combined **GLCM.** This combined GLCM is then normalised by dividing each element by the total number of pixel pairs, resulting in a matrix that represents the probability distribution of how often specific pairs of pixel intensities occur next to each other in the image (**Supplementary Figure 1(c)**).

From the normalised GLCM, various statistical features can be derived to quantitatively describe the texture of the image. In these calculations, ‘i’ and ‘j’ are coordinates of the co-occurrence matrix, and $\sigma$ and $\mu$ represent standard deviations and means for an angle $\Theta$. The textural features measured were:

1. Angular Second Moment

Angular second moment (ASM) is a measure of the ***global image homogeneity*** or uniformity of an image. In a homogeneous image, only a few dominant grey tones are present, meaning the image consists mostly of a single shade. This indicates a highly uniform texture such as that seen in normal aerated lung and will compute a high ASM value. Low ASM values suggests high variability in pixel intensities (more complex or rough surfaces) such as in heterogenous lung regions in pathology.

Mathematically, it is calculated as the sum of squares of entries within the GLCM, where $p\left( i,j \right)$ represents the probability of a pixel pair occurring at specific grey levels. A higher probability of pixel pairs p(i,j) repeating with an image leads to a higher f1 value.

$$f_{1}= \sum_{i} \sum_{j} \left\{ p\left( i,j \right) \right\}^{2}$$

1. Contrast

Contrast is a measure of local variations in an image i.e. a measure of the ***intensity difference*** between neighbouring pixels in an image. The larger the amount of local variation present in an image, the higher the contrast, suggesting sharp transitions or rough texture. Low contrast indicates mostly similar intensities as seen in smooth texture and uniform regions.

$f_{2}=\sum_{n=0}^{N_{g-1}} n^{2}\left\{ \begin{aligned} \sum_{i=1}^{Ng} \sum_{j=1}^{Ng} p(i,j) \\ \left| i-j \right|=n \end{aligned} \right\}$.

1. Correlation (Q-LUS_correlation_)

Correlation quantifies the relationship between neighbouring pixel intensities, assessing ***how consistently pixel values change over an image***. High correlation indicates pixel values follow a structured, predictable pattern whereas low correlation indicates pixel values are randomly distributed, indicating a disorganized or chaotic texture. An image can be highly correlated in one direction, and less so in another direction. Clinically, high correlation indicates a structured pleural line and smooth lung surface. The range of correlation values range from -1 to 1.

$f_{3}= \frac{\sum_{i} \sum_{j} \left( ij \right)p\left( i,j \right) - \mu_{x}\mu_{y}}{\sigma_{x}\sigma_{y}}$.

1. Inverse difference moment

Inverse difference moment (IDM) measures ***similarity of neighbouring pixels***. An image with smooth texture will have neighbouring pixels with similar intensity values. This results in a high IDM value as can be seen mathematically; the denominator penalizes large intensity differences. A low IDM indicates the presence of edges, sharp intensity variations or rough textures. Clinically, this could indicate pathological lung conditions where the pleural line is disrupted, or consolidation is present. The range of values IDM can take is 0 to 1, where a value of 1 indicates when pixel intensities are entirely the same such as in a smooth image.

$$f_{4}= \sum_{i} \sum_{j} \frac{1}{1+\left( i-j \right)^{2}}p\left( i,j \right).$$

1. Entropy

***Entropy measures texture randomness.*** If the GLCM is spread out, entropy is high meaning that the pattern is complex. A highly detailed or noisy image will have high entropy. Low entropy indicates the image has a more uniform and ordered structure with repetitive intensity patterns.

$$f_{5}= - \sum_{i} \sum_{j} p\left( i,j \right)\log\left( p \left( i,j \right) \right).$$

Overall, a smooth image is expected to have high ASM, low contrast, high Q-LUS_correlation_, high IDM and low entropy.

To understand how these textural features may appear in an image, we can refer to **Supplementary Figure 2.**

**
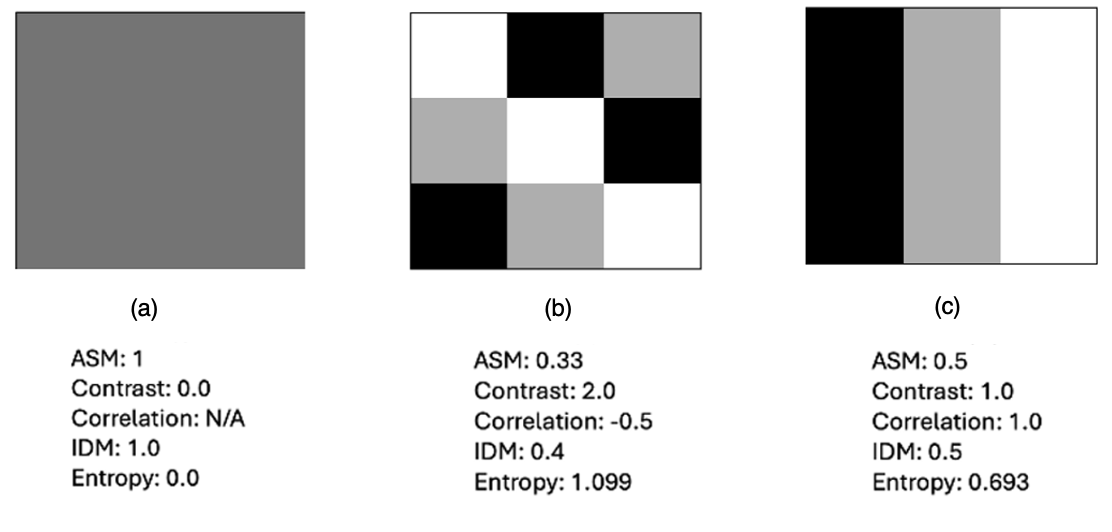
**

**Supplementary Figure 2.** Textural feature values for three 3×3 images, derived from GLCM analysis in the horizontal (left-to-right) direction.

**Supplementary Figure 2(a)** shows a completely uniform texture composed of a single grey level. As the image is fully homogeneous, the ASM and IDM reach a maximum of 1.0. There is no variation in intensity, so contrast is 0, Q-LUS_correlation_ is undefined due to zero variance, and entropy is 0, reflecting complete predictability.

In contrast, **Supplementary Figure 2 (b)** exhibits a highly disordered arrangement of pixel intensities. This image has a lower ASM (0.33) and IDM (0.4), indicating less uniformity. Greater variation in adjacent pixel intensities produces high contrast (2.0), and the lack of a consistent spatial pattern results in a moderate negative Q-LUS_correlation_ (-0.5). A broad distribution of grey levels leads to the highest entropy (1.099), indicating a complex, unpredictable texture.

**Supplementary Figure 2(c)** presents a structured gradient of pixel values from left-to-right. The image is more homogenous in the left-to-right direction than image B but less so than image A, resulting in a moderate ASM (0.5) and IDM (0.5) value. Horizontally, the stepwise changes in intensity give rise to moderate contrast (1.0). There is a strong Q-LUS_correlation_ (1.0) moving horizontally. The limited variety in transitions yields a lower entropy (0.693) compared to Figure 4B.


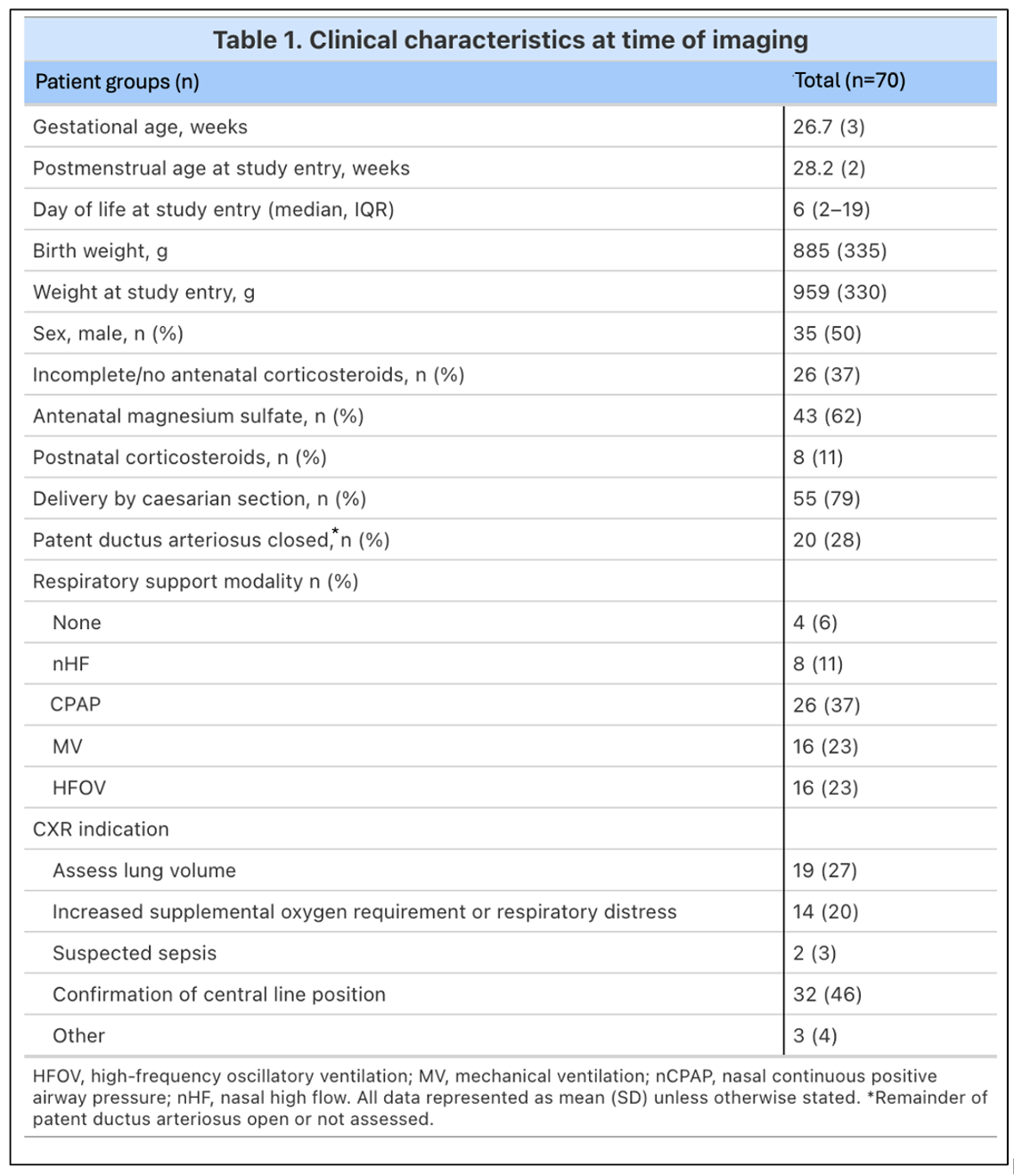


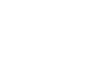
 **Supplementary Table 1.** Clinical characteristics at time of imaging.

|  | **Overall** | | **Venue 50** | | **Venue Go** | |
| --- | --- | --- | --- | --- | --- | --- |
| **Oxygenation** | **OSI**  **(95% CI)** | **S/F Ratio**  **(95% CI)** | **OSI**  **(95% CI)** | **S/F Ratio**  **(95% CI)** | **OSI**  **(95% CI)** | **S/F Ratio**  **(95% CI)** |
| **MGV** | **-0.46 (**-0.68,-0.23) | **0.38 (**0.16,0.61) | **-0.43 (**-0.69,-0.18) | **0.34 (**0.06,0.63) | **0.12** (-0.61,0.83) | **-0.24 (-0.85,0.37)** |
| **ASM** | **-0.17 (-0.45,0.10)** | **0.11 (-0.14,0.36)** | **-0.065(-0.39,0.26)** | **0.014 (-0.28,0.31)** | **-0.35 (-0.91,0.22)** | **0.30 (-0.27,0.85)** |
| **Contrast** | **-0.056 (-0.35,0.24)** | **0.073 (-0.16,0.31)** | **0.38 (**0.13,0.62) | **-0.31 (**-0.52,-0.10) | **-0.21 (-0.89,0.48)** | **0.32 (-0.25,0.89)** |
| **Correlation** | **0.48 (**0.25,0.71) | **-0.54 (**-0.74,-0.34) | **0.33 (**0.01,0.64) | **-0.45 (**-0.72,-0.18) | **0.65 (**0.14,0.94) | **-0.62 (**-1.05,0.25) |
| **IDM** | **0.084 (-0.22,0.39)** | **-0.088 (-0.34,0.16)** | **-0.33 (**-0.62,-0.03) | **0.27 (-0.0048,0.53)** | **0.25 (-0.48,0.96)** | **0.25 (-0.87,0.48)** |
| **Entropy** | **-0.31(**-0.58,-0.03) | **0.35 (**0.12,0.57) | **-0.099 (-0.43,0.25)** | **0.19 (-0.10,0.48)** | **-0.58 (**-1.0,-0.13) | **0.45 (-0.12, 0.83)** |

**Supplementary Table 2.** Spearman’s correlation coefficients and 95% confidence intervals (CI) for grey-level co-occurrence matrix (GLCM) features in relation to oxygen saturation index (OSI) and saturation to fraction of inspired oxygen (S/F) ratio. **All correlations are reported, including both statistically significant and non-significant results.** Statistically significant correlations (p < 0.01) are highlighted as fair (orange) or moderate (green), while non-significant results are shown without shading. ASM = angular second moment; IDM = inverse difference moment; MGV = mean gray value; NS = not significant.
